# Anti-Inflammatory Effects of Cannabigerol In Vitro and In Vivo Are Mediated Through the JAK/STAT/NFκB Signaling Pathway

**DOI:** 10.3390/cells14020083

**Published:** 2025-01-09

**Authors:** Ga Hee Jeong, Ki Chan Kim, Ji Hyun Lee

**Affiliations:** 1 Department of Medical Sciences, Graduate School of The Catholic University of Korea, Seoul, #222 Banpo-daero, Seocho-gu, Seoul 06591, Republic of Korea; 2 Department of Dermatology, Seoul St. Mary’s Hospital, College of Medicine, The Catholic University of Korea, Seoul, #222 Banpo-daero, Seocho-gu, Seoul 06591, Republic of Korea

**Keywords:** cannabigerol, Janus kinase-signal transducer and activator of transcription, atopic dermatitis

## Abstract

Cannabinoid compounds have potential as treatments for a variety of conditions, with cannabigerol (CBG) being known for its anti-inflammatory properties. In this study, we investigated the effects of CBG in a cellular model of 1-chloro-2,4-dinitrobenzene (DNCB)-induced atopic dermatitis (AD). In the cellular model, we confirmed the cytotoxicity of CBG and downregulated the expression of inflammatory markers *CCL26*, *IL1B*, *IL6*, and *TNF* (*p* < 0.001). In the mouse model, clinical, histological, and immunological changes were analyzed. The results showed that CBG improved dermatitis severity score, epidermal thickness, and mast cell count and reduced inflammatory cytokines (*Tslp*, *Il1b*, *Il4*, *Il6*, *Il13*, *Il17*, *Il18*, *Il22*, and *Il33*) by qRT-PCR (*p* < 0.001). Western blot results showed modulated changes in JAK1, JAK2, TYK2, STAT1, STAT2, STAT3, p-STAT3, STAT6, and p-STAT6 (*p* < 0.05). Subsequently, p-IκBα, NF-κB, and p-NF-κB signaling factors were also reduced (*p* < 0.05), with corresponding changes in skin barrier factors. The results of this study indicate that CBG effectively alleviates AD-like symptoms and suggest the potential of CBG as a therapeutic agent.

## 1. Introduction

Cannabinoid compounds have garnered significant attention for their therapeutic potential across various medical conditions. Cannabigerol (CBG), a plant-derived phytocannabinoid, is central to the biosynthetic pathway of the cannabis plant and serves as a precursor to major cannabinoids such as Δ-9-tetrahydrocannabinol (THC), Cannabidiol (CBD), and Cannabinol (CBN) [1]. CBG, a non-psychoactive cannabinoid, interacts with cannabinoid receptors in the body without inducing psychoactive effects [1], making it a promising candidate for therapeutic use.

Globally, approved cannabinoid medications include Sativex (THC Botanical Drug Substance) and Epidiolex (CBD), used for multiple sclerosis spasticity and epilepsy, respectively [2,3]. Recent studies suggest cannabinoids’ potential efficacy in treating inflammatory conditions [4,5,6], including COVID-19 [7,8,9,10], cancer [10,11], and skin disorders. Previous studies have shown that CBD has an alleviating effect in a mouse model of psoriasis, and in a large double-blind, randomized, placebo-controlled trial in patients with psoriasis, CBD ointment was shown to reduce severity scores [12,13]. There have also been reports that topical application of a mixture of ginger extract and CBD or a 1% CBD gel can alleviate symptoms of atopic dermatitis (AD) [14,15].

AD is a chronic inflammatory condition characterized by intense itching, erythema, edema, and impaired skin barrier function [16,17,18,19]. Its pathogenesis involves a complex interplay of genetic predisposition, environmental factors, and immune dysregulation. Central to its inflammatory responses are T-helper 2 cytokines such as IL-4 and IL-13, which contribute to skin barrier dysfunction and heightened immune responses [16,17,18]. Topical treatment is the preferred treatment for AD [20,21]. Traditionally formulated treatments for AD have increased the frequency of administration and other side effects when used over a long period of time [21]. Tacrolimus, a calcineurin inhibitor, has been reported to have some side effects such as itching, burning, and skin infections, and PDE4 inhibitors have side effects such as burning and tingling at the application site [21,22,23].

Previous studies have shown that CBG, in addition to its known role as an alpha2-adrenoceptor agonist [24], has analgesic effects in various pain models, is relatively safe with low toxicity, and is considered promising for developing transdermal administration for pain management [25]. It has also been shown to have potent antibacterial effects against *S. aureus* [26], which colonizes the skin of AD patients and contributes to the development and exacerbation of AD [27]. In particular, CBG has shown anti-inflammatory properties by regulating cytokine and chemokine production in keratinocytes [28,29]. Unlike THC and CBD, which are already approved as medicines, research into CBG is still in its early stages. Therefore, we used AD cells and mouse models to investigate its potential effects on AD.

This study examines the potential of CBG as a treatment for AD by investigating its effects on the Janus kinase-signal transducer and activator of transcription (JAK-STAT) pathway, a key regulator of cytokine signaling and immune responses in skin diseases. A comprehensive evaluation of clinical symptoms and molecular biomarkers may establish CBG as a promising topical therapy for inflammatory skin diseases.

## 2. Materials and Methods

### 2.1. Materials

The cannabigerol isolate utilized in this study was supplied by Yuhan Care Co., Ltd. (Seoul, Republic of Korea). Authorization for the use of this material was granted by the Seoul Regional Food and Drug Administration under the Narcotics Handler Permit—Narcotics Researcher (No. 2018).

### 2.2. Cell Culture and Reagents

HaCaT cells, a spontaneously immortalized keratinocyte cell line derived from the human epidermis, were acquired from the American Type Culture Collection (Manassas, VA, USA). Cells were cultured in Dulbecco’s Modified Eagle’s Medium (DMEM) with high glucose (Gibco, Carlsbad, CA, USA), supplemented with 1% penicillin/streptomycin (Gibco) and 10% fetal bovine serum (Gibco), under a humidified atmosphere of 5% CO_2_ at 37 °C. Recombinant Human IL-4 and IL-13 were purchased from PEPROTECH. A stock solution of cannabigerol was prepared in DMSO at a concentration of 100 mM.

### 2.3. Cell Viability Assay

HaCaT cells were seeded at a density of 1 × 10^4^ cells/well in a 96-well plate. After 24 h, the medium was replaced with serum-free medium, and the cells were treated with cannabigerol (1–200 μM) for 24 h. Cell viability was assessed using the MTT assay. MTT solution (100 µL, 5 mg/mL) was added to each well and incubated for 2 h at 37 °C. Subsequently, the supernatant was removed, and the MTT-formazan crystals formed by metabolically active cells were dissolved in 80 µL of isopropanol (Merk Millipore, Burlington, MA, USA). Absorbance was measured at 540 nm using a VersaMax microplate reader (Molecular Devices, San Jose, CA, USA).

### 2.4. Quantitative Real-Time PCR

Dorsal skin tissue was collected using a 5 mm biopsy punch. Each of the keratinocyte and mouse skin RNAs was extracted using TRIzol reagent (Invitrogen, Carlsbad, CA, USA). RNA quantity was measured with a NanoDrop spectrophotometer (Thermo Fisher Scientific, Waltham, MA, USA). Quantitative real-time PCR was conducted on a CFX-96 thermocycler (Bio-Rad, Hercules, CA, USA) using primers mixed with Power SYBR^®^ Green PCR Master Mix (Takara Biomedical Inc., Shiga, Japan). PCR conditions and the 2−ΔΔCT method of analysis were referenced as follows [30,31], with data normalized to *GAPDH* or *Actb*. Primer sequences are listed in Table 1.

### 2.5. Animal Study

Male BALB/C mice, aged six weeks (16–20 g), were purchased from Japan SLC, Inc. (Shizuoka, Japan). A total of 25 mice were used in this study, randomly assigned into five groups with five mice per group.

Mice were housed under standardized conditions with a 12 h light/dark cycle, at a constant temperature of 23 ± 3 °C, and 50 ± 10% humidity. They had ad libitum access to autoclaved R/O water and sterilized feed (1310, Altromin, Lage, Germany). Environmental enrichment, including mouse houses and nesting materials, was provided to enhance animal welfare. Isoflurane anesthesia (Ifran Solution, Hana Pharm. Co., Ltd., Seoul, Republic of Korea) was administered during procedures such as shaving, substance administration, and clinical evaluations to minimize animal distress. For the induction of atopic dermatitis (AD), 1-chloro-2,4-dinitrobenzene (DNCB; Sigma-Aldrich, St. Louis, MO, USA) was employed. The DNCB solution was prepared at concentrations of 1% and 0.4% in an acetone/olive oil suspension (4:1). During the first week, mice were sensitized by applying 1% DNCB to their abdomens twice. Prior to the challenge phase, dorsal hair was removed using animal clippers and hair removal cream. In the challenge phase, 0.4% DNCB was applied twice weekly for two weeks. The vehicle (acetone/olive oil 4:1), 0.1 mg/kg and 1 mg/kg of cannabigerol (acetone/olive oil 4:1), and 0.03% tacrolimus ointment were applied thrice weekly during the same period. All animal procedures were conducted in accordance with the Laboratory Animals Welfare Act, the Guide for the Care and Use of Laboratory Animals, and the Guidelines and Policies for Rodent Experiments, as approved by the Institutional Animal Care and Use Committee of the School of Medicine, The Catholic University of Korea (approval no. CUMS-2023-0142-01).

### 2.6. Evaluation of Skin Lesions

During the challenge phase, the severity of skin lesions on the back and ears of each mouse was monitored. The assessment was performed by three independent clinicians, evaluating three parameters: (1) erythema/hemorrhage, (2) scarring/dryness, and (3) edema. Each parameter was scored on a scale of 0 to 3, where 0 indicated no symptoms, 1 mild, 2 moderate, and 3 severe symptoms. Evaluations were conducted every three days, totaling six assessments over the two-week challenge period [31].

### 2.7. Histological Analysis

Tissue samples were fixed in 4% formaldehyde, embedded in paraffin, and sectioned at 5 μm thickness. Sections were stained with hematoxylin and eosin for general histology, and toluidine blue solution was used for mast cell staining. Stained mast cells were counted, and images were captured using a DM2500 LED light microscope (Leica Microsystems, Wetzlar, Germany). Epidermal hyperplasia was assessed by measuring epidermal thickness with Leica Application Suite X software (version 3.7.1.21655) (Leica Microsystems, Wetzlar, Germany).

### 2.8. Western Blot

Dorsal skin samples were stored at −80 °C, and protein lysates were prepared using T-PER lysis buffer with a protease inhibitor cocktail (Thermo Fisher Scientific, Waltham, MA, USA). Protein concentrations were determined with the BCA Protein Assay Kit II (Thermo Fisher Scientific, Waltham, MA, USA), and equal amounts (20 or 40 μg) were separated by sodium dodecyl sulfate-polyacrylamide gel electrophoresis (SDS-PAGE) and transferred to polyvinylidene fluoride (PVDF) membranes (Millipore Sigma, St. Louis, MO, USA). Membranes were blocked with 5% skim milk or 5% bovine serum albumin (BSA) in Tris-buffered saline with 0.1% Tween 20 (TBS-T) for 1 h at room temperature, followed by overnight incubation at 4 °C with primary antibodies. After TBS-T washes, horseradish peroxidase (HRP)-conjugated second antibodies (GeneTex, Irvine, CA, USA) were applied for 2 h at room temperature. Protein bands were visualized with ECL substrate (Thermo Fisher Scientific) using an Amersham™ Imager 600 (GE Healthcare, Chicago, IL, USA), and band intensities were quantified with ImageJ software (version 1.54f; Wayne Rasband and contributors National Institute of Health, Bethesda, MD, USA, http://imagej.org, accessed on 9 December 2023). The primary antibodies used included β-actin (1:2500, #3700), JAK1 (1:400, #3344), JAK2 (1:1000, #3230), TYK2 (1:500, sc-5271), STAT1 (1:1000, #14994), STAT2 (1:1000, #72604), STAT3 (1:1000, #9139), phospho-STAT3 (1:1000, #9145), STAT6 (1:1000, #9362), phospho-STAT6 (1:1000, #56554), IκBα (1:1000, #9242), phospho-IκBα (1:1000, #9246), NF-κB p65 (1:1000, #3033), and phospho-NF-κB p65 (1:1000, #8242) (Cell Signaling, San Diego, CA, USA).

### 2.9. Statistical Analysis

Statistical analysis was performed using one-way analysis of variance (ANOVA) followed by Tukey’s multiple-comparisons test. Unpaired *t*-tests were used for group comparisons. Graphs were created using GraphPad Prism 5 (GraphPad Software, La Jolla, CA, USA), and all data are presented as the mean ± standard error of the mean (SEM). Statistical significance was defined as *p* < 0.05 (* *p* < 0.05; ** *p* < 0.01; *** *p* < 0.001).

## 3. Results

### 3.1. CBG Suppresses the Expression of Inflammatory Cytokines and Chemokines in Keratinocytes Stimulated with IL-4 and IL-13

**Figure 1:**
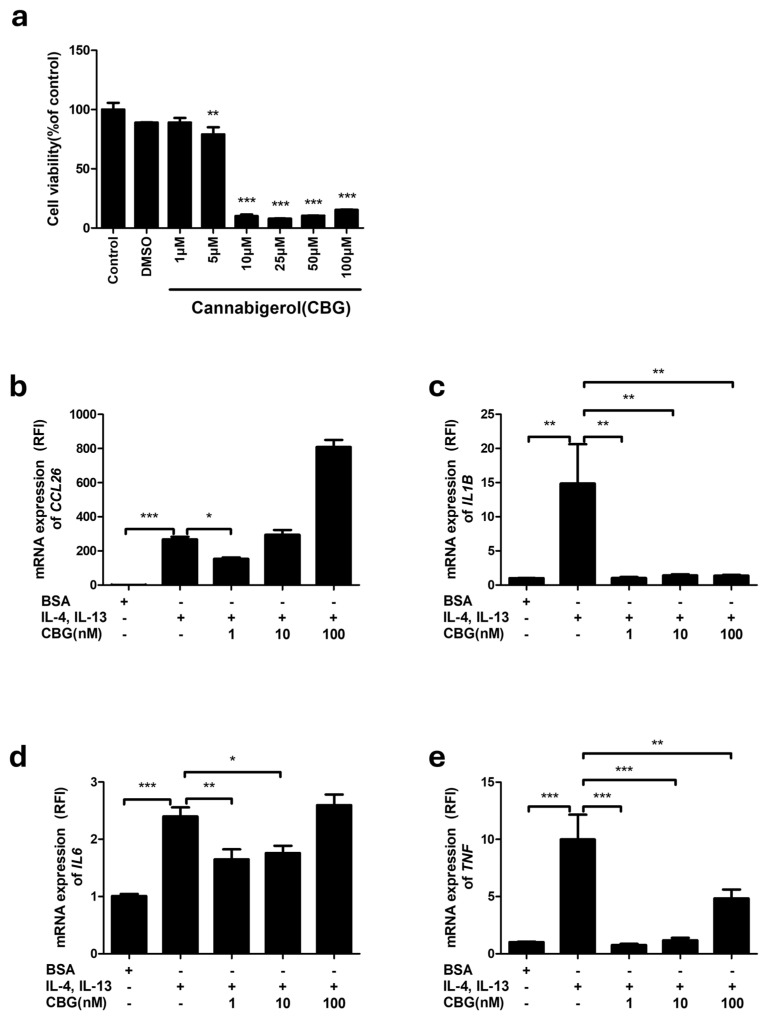
Effect of CBG treatment against IL-4 and IL-13 induction. (**a**) CBG toxicity in HaCaT keratinocytes. assessed by MTT assay and expressed as a percentage relative to Control cells. Data represents the mean ± SEM from three independent experiments. ** *p* < 0.01 *** *p* < 0.001 compared with Control cells. (**b**–**e**) HaCaT cells were treated with 50 ng each of IL-4 and IL-13 for 18 h, followed by CBG treatment at indicated concentrations for 6 h. Cytokine mRNA levels were measured and expressed as relative fold increases compared to CBG controls. Data represents the mean ± SEM from three independent experiments. Data compared among multiple groups were analyzed using one-way ANOVA. * *p* < 0.5, ** *p* < 0.01, *** *p* < 0.001 compared with BSA control or IL-4, IL-13 treated cells. CBG, cannabigerol; SEM, standard error of the mean; ANOVA, analysis of variance.

In a study investigating the anti-inflammatory effects of CBG on keratinocytes stimulated with IL-4 and IL-13, cell viability was first assessed to determine the non-toxic concentrations of CBG. Using an MTT assay, HaCaT cells were treated with CBG at concentrations ranging from 1 to 100 μM for 24 h. As shown in Figure 1a, cell viability was evaluated with the Control cells set at 100%. The results indicated that CBG exhibited cytotoxicity at concentrations of 5 μM and higher, but not at 1 μM. Specifically, cell viability was 89% for both DMSO and 1 μM CBG, while it dropped significantly at higher concentrations: 79% for 5 μM, 10% for 10 μM, 7.8% for 25 μM, 10.3% for 50 μM, and 15.2% for 100 μM CBG (Figure 1a).

To induce an inflammatory environment in HaCaT cells, we used IL-4 and IL-13 at a concentration of 50 ng/μL, with 0.1% BSA as a control, treating for a total of 18 h. Then, we treated with 1, 10, 100, and 1000 nM of CBG for 6 h. We performed quantitative real-time PCR (qRT-PCR) to examine the mRNA expression levels of inflammatory cytokines and chemokines. Upon stimulation with IL-4 and IL-13, the mRNA expression levels of *CCL26*, *IL1B*, *IL6*, and *TNF* were significantly increased compared to the BSA group (b) (Figure 1b–e). *CCL26* was significantly decreased in the 1 nM CBG group. No significant difference was observed at 10 nM, while a significant increase was noted in the 100 nM group compared to the IL-4 and IL-13 group. Significant differences were observed between the 1 nM and 10 nM groups, as well as between the 10 nM and 100 nM groups (Figure 1b). *IL1B* was significantly decreased in the 1, 10, 100, and 1000 nM groups compared to the IL-4 and IL-13 group (Figure 1c). *IL6* was significantly decreased in the 1, 10, and 1000 nM CBG groups compared to the IL-4 and IL-13 group, with no significant difference observed at 100 nM. Significant differences were noted between the 10 nM and 100 nM groups, as well as between the 100 nM and 1000 nM groups (Figure 1d). *TNF* was significantly decreased in the 1, 10, 100, and 1000 nM CBG groups compared to the IL-4 and IL-13 group, with significant differences observed only between the 10 nM and 100 nM groups (Figure 1e).

These results indicate that all cytokines and chemokines were significantly increased in the inflammatory IL-4 and IL-13 treatment group compared to the BSA group (Figure 1b–e). Interestingly, all cytokine and chemokine levels were reduced at the lowest concentration of 1 nM CBG. Additionally, *IL1B* and *TNF* levels demonstrate consistent decreases across all concentrations of CBG treatment (Figure 1c,e).

### 3.2. Topical CBG Treatment Alleviates Clinical Symptoms Such as Erythema and Scaling in an Atopic Dermatitis Animal Model

**Figure 2:**
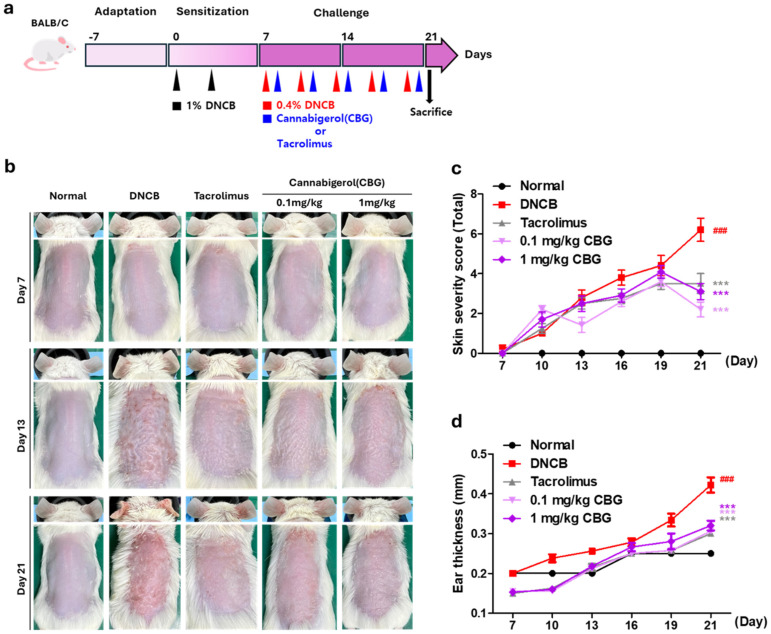
CBG alleviated AD on clinical evaluation. (**a**) To evaluate the efficacy of topical CBG treatment in an AD-like environment, an experimental schedule was established as shown in the accompanying figure. The treatment phases and their frequency are indicated by arrows. The experiment includes abdominal sensitization with 1% DNCB (black), maintenance of the AD environment during the challenge period with 0.4% DNCB (red), and application of vehicles, tacrolimus, and CBG (blue). (**b**) Clinical results at the end of a 2-week challenge in an animal model of AD. Each image shows normal skin or skin treated with vehicle (acetone: olive oil suspension), tacrolimus, 0.1 mg/kg, and 1 mg/kg CBG on days 7, 13, and 21. (**c**) Skin severity score, encompassing erythema/hemorrhage, scaling/dryness, and edema, is shown for Days 7–21. (**d**) Ear thickness assessed results on Day 7–21. Data represents the mean ± SEM (*n* = 5). Data compared among multiple groups were analyzed using one-way ANOVA. ### *p* < 0.001 compared to Normal group, *** *p* < 0.001 compared to DNCB group. CBG, cannabigerol; AD, atopic dermatitis; DNCB, 1-chloro-2,4-dinitrobenzene; SEM, standard error of the mean; ANOVA, analysis of variance.

To investigate the therapeutic effects of CBG on AD, skin inflammation was induced in BALB/c mice using DNCB following a week sensitization period (Figure 2a). The AD model induced by DNCB was treated with vehicle (acetone: olive oil 4:1), tacrolimus, 0.1 mg/kg CBG, or 1 mg/kg CBG, administered 5 times over 2 weeks (every 3 days) (Figure 2a). Photographs were taken every 7 days from the start of induction for comparison (Figure 2b). On day 21, the skin severity score for erythema/hemorrhage, scaling/dryness, and edema in the DNCB group was significantly elevated at 6.2 (±0.58) compared to the normal group (*p* < 0.001). Ear thickness in the DNCB group was also significantly increased to 0.42 mm (*p* < 0.001) (Figure 2c,d). In contrast, the 0.1 mg/kg CBG group exhibited decreases in skin severity scores and ear thicknesses of 2.2 (±0.374) and 0.30 mm, while the 1 mg/kg CBG group showed scores of 3.1 (±0.4) and 0.32 mm (Figure 2c,d). Both the 0.1 mg/kg and 1 mg/kg CBG groups demonstrated significant decreases in skin severity scores and ear thickness compared to the DNCB group (*p* < 0.001) (Figure 2c,d).

### 3.3. Cannabigerol Reduces Epidermal Hyperplasia and Mast Cell Infiltration in DNCB-Induced Mice (Atopic Dermatitis Animal Model)

**Figure 3:**
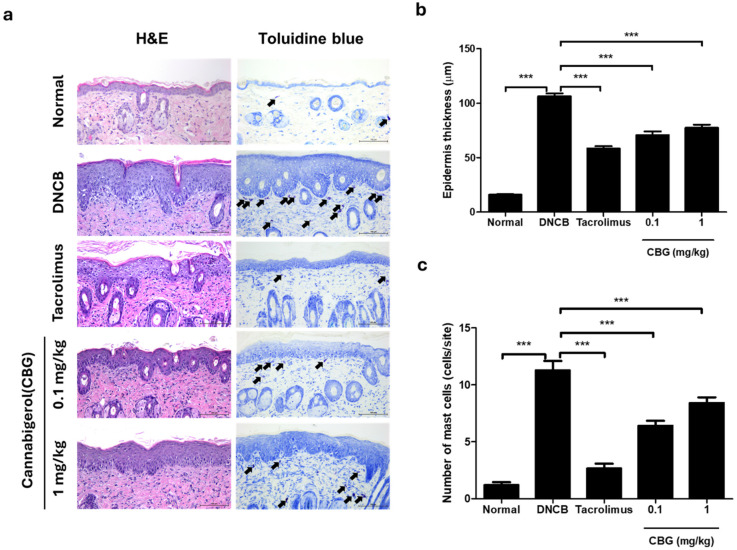
CBG reduced skin thickness and mast cell count. (**a**) Representative images from H&E and toluidine blue staining of dorsal skin tissues from mice after the challenge period. Histological features of dorsal skin in BALB/c mice are depicted. Mast cells (purple) in the dermis are indicated and marked with black arrows. Original magnification = ×200, scale bar = 100 μm. (**b**) Epidermal thickness changes for each group. (**c**) The number of mast cells counted and compared across each group. Data represents the mean ± SEM. Data compared among multiple groups were analyzed using one-way ANOVA. *** *p* < 0.001 compared to Normal or DNCB group. CBG, cannabigerol; H&E, Hematoxylin and Eosin; DNCB, 1-chloro-2,4-dinitrobenzene; SEM, standard error of the mean; ANOVA, analysis of variance.

The immunological characteristics of AD patients include the infiltration of inflammatory cells and mast cells around the lesions and epidermal hyperplasia. The histopathological characteristics of dorsal skin tissues were analyzed by hematoxylin-eosin (H&E) staining and toluidine blue staining (Figure 3a). In H&E staining, the DNCB group showed epithelial hyperplasia and keratinization compared to the normal group (*p* < 0.001), and 0.1 mg/kg CBG and 1 mg/kg CBG treatment significantly reduced epidermal thickness (*p* < 0.001) (Figure 3b). Quantification of mast cell number by toluidine blue staining showed that the number of mast cells was significantly higher in the DNCB group (*p* < 0.001), but 0.1 mg/kg CBG and 1 mg/kg CBG treatment significantly reduced the number of mast cells (*p* < 0.001) (Figure 3c).

### 3.4. Cannabigerol Attenuates Allergen-Induced Inflammatory Cytokine Secretion and Modulates mRNA Expression

**Figure 4:**
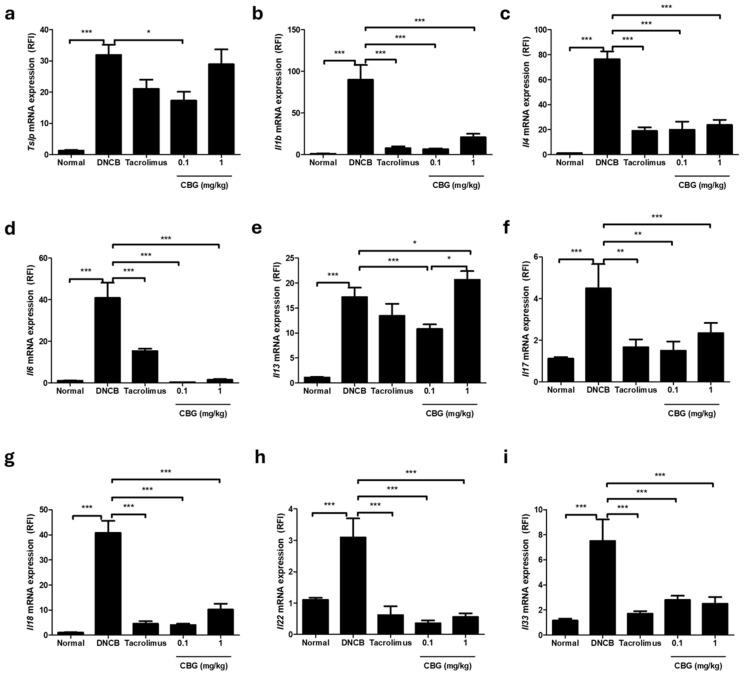
Effects of CBG on cytokine expression in a DNCB-induced AD mouse model. Changes in mRNA expression levels of (**a**) *Tslp*, (**b**) *Il1b*, (**c**) *Il4*, (**d**) *Il6*, (**e**) *Il13*, (**f**) *Il17*, (**g**) *Il18*, (**h**) *Il22*, (**i**) *Il33* in dorsal skin tissue. Total RNA was extracted and analyzed using qRT-PCR (*n* = 5/group). Gene expression levels were normalized to *Actb*. The qRT-PCR was duplicated, and data were analyzed by CFX Manager software (version 3.0) (Bio-Rad Laboratories, Hercules, CA, USA). Data represents the mean ± SEM. Data compared among multiple groups were analyzed using one-way ANOVA. * *p* < 0.05, ** *p* < 0.01, *** *p* < 0.001 compared to Normal or DNCB group. CBG, cannabigerol; DNCB, 1-chloro-2,4-dinitrobenzene; AD, atopic dermatitis; SEM, standard error of the mean.

To investigate the impact of CBG on AD development, we analyzed the expression levels of skin inflammation signaling genes using (qRT-PCR). In the DNCB-induced AD model, the mRNA expression of inflammatory cytokines *Tslp*, *Il1b*, *Il4*, *Il6*, *Il13*, *Il17*, *Il18*, *Il22*, and *Il33* was significantly increased (*p* < 0.001) (Figure 4). Following 2 weeks of treatment, tacrolimus significantly reduced the expression of *Il1b*, *Il4*, *Il6*, *Il17*, *Il18*, *Il22,* and *Il33*. Additionally, various concentrations of CBG were able to reduce the mRNA expression of inflammatory markers *Tslp*, *Il1b*, *Il4*, *Il6*, *Il13*, *Il17*, *Il18*, *Il22,* and *Il33* to varying degrees (Figure 4). Our results demonstrate that CBG can downregulate inflammatory cytokines involved in allergen-induced skin inflammation signaling.

### 3.5. Effects of Cannabigerol on Inflammatory Signaling Pathways in Atopic Dermatitis

**Figure 5:**
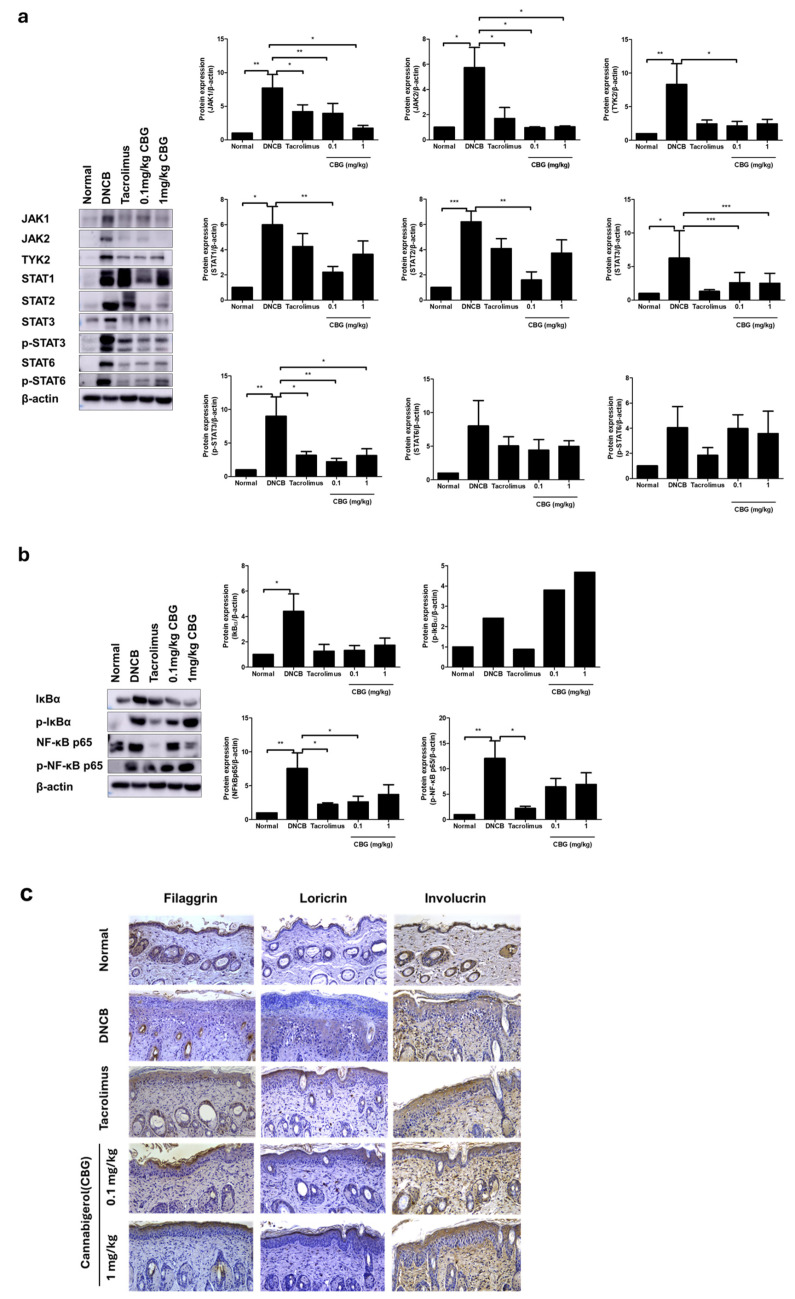
Effects of CBG on JAK/STAT, NF-κB signaling pathway, and skin barrier proteins in a DNCB-induced AD mouse model. Changes in protein expression levels of (**a**) JAK/STAT pathway family proteins. (**b**) NF-κB pathway family proteins (**c**) skin barrier protein in dorsal skin tissue. Original magnification = ×200, scale bar = 100 μm Immunoblotting intensities were calculated with ImageJ software (version 1.54f). Data represents the mean ± SEM. Data compared among multiple groups were analyzed using one-way ANOVA. * *p* < 0.05, ** *p* < 0.01, *** *p* < 0.001 compared to normal or DNCB group. CBG, cannabigerol; DNCB, 1-chloro-2,4-dinitrobenzene; AD, atopic dermatitis; SEM, standard error of the mean; ANOVA, analysis of variance.

In our study, we observed a reduction in inflammatory cytokines following CBG treatment in an AD mouse model. This led us to investigate whether CBG affects the JAK-STAT signaling pathway, which plays a crucial role in regulating inflammatory cytokines and T-cell responses in AD. Our results indicated that in the mouse model, key JAKs involved in IL-4 and IL-13 signaling, namely JAK1, JAK2, and TYK2, were increased (*p* < 0.05) (Figure 5a). Additionally, the activation of the JAK family was associated with elevated signaling of STAT1, STAT2, STAT3, STAT6, p-STAT3, and p-STAT6 (Figure 5a). CBG treatment was found to decrease the activity of the JAK-STAT family. Notably, the group treated with 0.1 mg/kg CBG exhibited a significant reduction in the activity of all examined signaling pathways (JAK1, JAK2, TYK2, STAT1, STAT2, STA3, p-STAT3, STAT6, p-STAT6) (*p* < 0.05) (Figure 5a). We also examined the NF-κB inflammatory signaling pathways, which can be activated by receptors such as IL-1 and TNF-α. IκBα, p-IκBα, NF-κB, andp-NF-κB levels were increased in the DNCB group (*p* < 0.05) (Figure 5b). CBG treatment resulted in decreased levels of p-IκBα, NF-κB, and p-NF-κB proteins compared to the DNCB group (*p* < 0.05) (Figure 5b). Consistent with the above results, we observed that the expressions of filaggrin, loricrin, and involucrin, proteins that represent the skin barrier, were restored in the CBG-treated group compared to the DNCB group (Figure 5c).

## 4. Discussion

In this study, we investigated the effects of CBG on AD using both in vitro and in vivo models. Inflammatory cytokines and chemokines such as *IL1B*, *IL6*, *TNF*, and *CCL26* were increased in keratinocytes stimulated with IL-4 and IL-13 (Figure 1). AD progression is influenced by IL-1 and IL-6, which are primarily secreted by epithelial and immune cells in AD lesions [32]. Keratinocytes express IL-4 and IL-13 receptors and produce the eosinophil chemokine CCL26 in response to IL-4 and IL-13. IL-4 also enhances the action of TNF-α, recruiting additional T cells to inflamed skin. CBG at concentrations of 1 nM was observed to significantly reduce the levels of cytokines and chemokines that are characteristic of AD, including *IL1B*, *IL6*, *TNF*, and *CCL26* (Figure 1).

The DNCB-induced AD mouse group had increased skin severity scores, ear and epidermal thickening, excessive keratin production, and an increased number of mast cells in the dermis (Figure 2 and Figure 3). Mast cells play a crucial role in allergic responses by synthesizing and releasing cytokines that promote inflammation and facilitate the infiltration of inflammatory cells into the skin [32]. CBG treatment significantly reduces symptoms such as ear and epidermal thickening, excessive keratinization, and mast cell infiltration into the dermis caused by DNCB application (Figure 2 and Figure 3). In addition, repeated hapten exposure, such as with DNCB, is known to shift immune responses from a Th1 to a Th2 profile, resulting in AD-like dermatitis [33,34,35].

In this study, topical CBG treatment reduced levels of *Tslp*, *Il1b*, *Il4*, *Il6*, *Il13*, *Il17*, *Il18*, *Il22*, and *Il33* induced by DNCB. Additionally, in both acute and chronic AD-like environments, the mRNA expression of Th1 and Th2 cytokines related to AD lesions was significantly reduced. Upon disruption of the epithelial barrier, TSLP activates immune cells, including mast cells within skin lesions, and acts as an important mediator of type 2 immune responses and a promoter of Th2 cell-mediated diseases [36,37,38]. IL-33, a cytokine produced by epithelial cells, can polarize Th2 responses and exacerbate eczema when upregulated [37,38]. IL-1 plays a central role in inflammatory initiation and is significantly upregulated in AD patients, especially those with filaggrin mutations.

The JAK-STAT signaling pathway is crucial for regulating cytokines involved in cell proliferation, homeostasis, and immune modulation. Dysregulation of this pathway, primarily mediated by JAK1 with contributions from JAK2 and TYK2, plays a key role in the pathogenesis of AD [39,40,41]. In the atopic environment, a dose-dependent reduction in the expression levels of JAK1, JAK2, TYK2, STAT3, p-STAT3, STAT6, and p-STAT6—excluding STAT1 and STAT2—has been observed (Figure 5).

It has been demonstrated that cannabis can suppress antitumor immunity by inhibiting JAK/STAT signaling in T cells through the activation of cannabinoid receptor 2 (CNR2) [42,43]. Cannabinoids, including CBG, may modulate immune responses via the JAK/STAT pathway. While primarily studied in cancer, this mechanism suggests potential applications in inflammatory diseases like AD, as seen with CBD in immune modulation [44]. Their study highlights that cannabinoids can influence immune responses by modulating cytokine production and signaling pathways, including the JAK-STAT pathway. This suggests that CBG, which shares structural similarities with CBD, might exert similar effects, potentially reducing inflammation in AD by modulating JAK-STAT signaling.

The crosstalk between NF-κB and STAT3, driven by IL-6 production, is essential in sustaining inflammation and promoting disease progression [45,46]. Since CBG has been shown to inhibit NF-κB activation, its anti-inflammatory effects could extend to the JAK-STAT pathway by reducing IL-6 levels and subsequent STAT3 activation. This dual inhibition of NF-κB and JAK-STAT signaling highlights CBGs comprehensive anti-inflammatory potential in AD.

Acetylsalicylic acid (also known as aspirin), which primarily inhibits the cyclooxygenase (COX) pathway and inhibits IκB kinase activity in mast cells [47], has been recommended as a topical immunomodulator for AD and as a therapy to relieve the application site erythema and burning that occur as side effects of tacrolimus [48]. However, in patients with AD who are prone to respiratory disease [49] or in children, the efficacy of aspirin may be limited, as observed in aspirin-exacerbated respiratory disease (AERD) [50]. In contrast, CBG targets both the JAK-STAT and NF-κB pathways, demonstrating a broader anti-inflammatory spectrum. This dual action positions CBG as a more comprehensive treatment option for inflammatory conditions such as AD with chronic inflammation. Despite these results, there are limitations to the use of CBG as a treatment for AD. While preclinical studies have highlighted its potential to modulate inflammatory pathways and restore skin barrier integrity, the lack of human clinical trials limits our understanding of its true therapeutic potential. Issues such as CBG formulation, dose optimization and standardization, and long-term safety need to be addressed. Addressing the limitations of regulatory barriers surrounding the use of cannabinoids will also be important in realizing the full potential of CBG as a treatment for AD.

In AD, inflammatory responses are often driven by cytokines such as IL-4, IL-13, and IL-5, which activate the JAK-STAT pathway [41]. These cytokines also downregulate key skin barrier proteins, including loricrin and involucrin, exacerbating barrier dysfunction and inflammation [41]. CBG has been shown to inhibit the production of pro-inflammatory cytokines such as IL-4 and IL-13, which are key drivers of type 2 inflammation in AD. Additionally, CBG reduces the expression of inflammatory cytokines like TNF-α and IL-1 through NF-κB inhibition, further supporting its anti-inflammatory role and its ability to restore skin barrier integrity. Inhibition of NF-κB by CBG can reduce the expression of inflammatory cytokines like TNF-α and IL-1, which are implicated in the downregulation of loricrin and involucrin in keratinocytes. This dual action on both the JAK-STAT and NF-κB pathways suggests that CBG may offer a comprehensive approach to mitigating the inflammatory processes in AD while simultaneously enhancing skin barrier function.

Most of the pharmacological effects of CBG are attributed to the activation of CB1 and CB2 receptors. However, evidence also suggests that the anti-inflammatory effects of these cannabinoids are induced through the activation of peroxisome proliferator-activated receptor gamma (PPAR-γ) [51,52]. PPAR-γ agonists inhibit the synthesis of inflammatory cytokines, such as TNF-α, IL-1β, and IL-6, in monocytes and suppress macrophage activation in vitro. CBG appears to inhibit the p65 NF-κB pathway activated through IL-1β and TNF-α receptors. In the AD mouse model, we observed an increase in IκBα, p-IκBα, NF-κB, and p-NF-κB in this pathway (Figure 5). Following CBG treatment, levels of p-IκBα, NF-κB, and p-NF-κB were reduced compared to the DNCB group (Figure 5). These findings, along with our study, suggest that the ability of CBG to inhibit JAK-STAT signaling may contribute to reducing NF-κB activation, thereby alleviating inflammation and symptoms in diseases such as AD. Further studies are needed to directly evaluate the effect of CBG on JAK-STAT signaling in AD.

The inflammatory response in AD is often driven by the activation of the JAK-STAT pathway, particularly through cytokines such as IL-4, IL-13, and IL-5 [41]. These cytokines are known to downregulate the expression of crucial skin barrier proteins like loricrin and involucrin, leading to further deterioration of the barrier. Moreover, the overactivation of the JAK-STAT pathway contributes to chronic inflammation and exacerbates AD symptoms [41]. CBGs anti-inflammatory properties can counteract this by modulating the JAK-STAT pathway [19]. CBG has been shown to inhibit the production of pro-inflammatory cytokines such as IL-4 and IL-13, which are key drivers of type 2 inflammation in AD (Figure 4). By reducing the activity of these cytokines, CBG can help decrease the inflammatory response and promote the expression of skin barrier proteins. Additionally, CBGs impact on the NF-κB pathway, another critical mediator of inflammation, further supports its anti-inflammatory role. Inhibition of NF-κB by CBG can reduce the expression of inflammatory cytokines like TNF-α and IL-1, which are implicated in the downregulation of loricrin and involucrin in keratinocytes. This dual action on both the JAK-STAT and NF-κB pathways suggests that CBG may offer a comprehensive approach to mitigating the inflammatory processes in AD while simultaneously enhancing skin barrier function.

One of the primary issues in AD is the disruption of the skin barrier, which is crucial for maintaining hydration and protecting against environmental irritants and allergens. The integrity of the skin barrier depends on the proper expression of structural proteins like filaggrin, loricrin, and involucrin [19,53,54]. Studies have shown that the knockdown of filaggrin in keratinocytes leads to reduced levels of these proteins, weakening the barrier and increasing susceptibility to irritants [53]. CBG has been shown to have protective effects on the skin barrier. For instance, in studies involving normal human epidermal keratinocytes (NHEKs) and human dermal fibroblasts (HDFs), CBG demonstrated superior performance over CBD in targeting genes related to collagen and elastin production. These proteins are critical for maintaining the structural integrity and elasticity of the skin. By enhancing the production of these components, CBG may help restore and strengthen the skin barrier in AD patients.

The clinical potential of CBG in AD management is further supported by recent studies that have demonstrated its effectiveness in reducing skin inflammation [6,55] and improving barrier function [56,57]. For example, a clinical trial involving a 0.1% CBG serum showed significant improvements in skin barrier function, including reductions in transepidermal water loss (TEWL) and skin redness [57]. These findings suggest that CBG can be a valuable addition to the therapeutic arsenal against AD, particularly for patients who struggle with chronic inflammation and barrier dysfunction.

## 5. Conclusions

**Figure 6:**
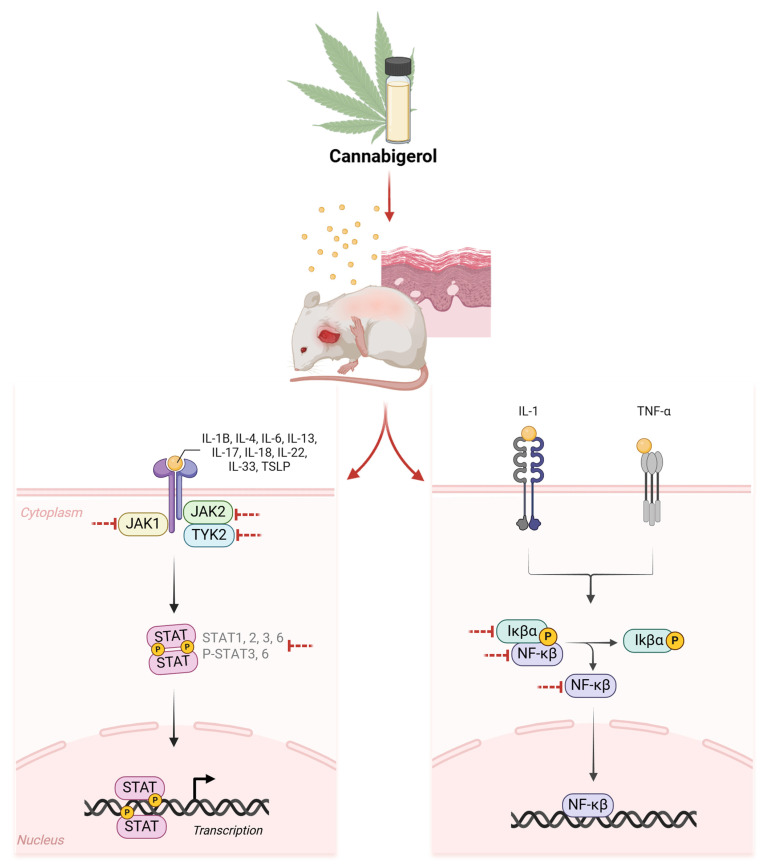
Effects of CBG on AD models. In the DNCB-induced AD model, the JAK/STAT signaling and NF-κB signaling pathways are activated by the combination of various inflammatory cytokines that have been found to have therapeutic effects on AD by modulating the signaling of these pathways. CBG, cannabigerol; AD, atopic dermatitis; DNCB, 1-chloro-2,4-dinitrobenzene.

In conclusion, our study demonstrates the therapeutic benefits of CBG in AD models. CBG inhibited some of the inflammatory cytokines and chemokines in AD cell models. In the mouse model, it inhibited inflammatory cytokines and JAK/STAT signaling (Figure 6), which was accompanied by improvement in histological assessments, including skin clinical scores. These results suggest that CBG may also affect the integrity of the skin barrier, including NF-κB signaling, and suggest that CBG is a potential treatment for AD.

## Figures and Tables

**Table 1:** Primer sequences for quantitative real-time PCR amplifications.

Host	Target	Sequence (5′-3′)	
		Forward	Reverse
human	*CCL26*	AACTCCGAAACAATTGTGACTCAGCTG	GTAACTCTGGGAGGAAACACCCTCTCC
	*IL1B*	ATGATGGCTTATTACAGTGGCAA	GTCGGAGATTCGTAGCTGGA
	*IL6*	GGCACTGGCAGAAAACAACC	GCAAGTCTCCTCATTGAATCC
	*TNF*	AACGGAGCTGAACAATAGGC	GGGCGATTACAGACACAACT
	*GAPDH*	GAAGGTGAAGGTCGGAGTCAA	GCTCCTGGAAGATGGTGATG
mouse	*Tslp*	AAAGGGGCTAAGTTCGAGCA	AGGGCTTCTCTTGTTCTCCG
	*Il1b*	TGCCACCTTTTGACAGTGAT	AGTGATACTGCCTGCCTGAA
	*Il4*	TCTCGAATGTACCAGGAGCCATATC	AGCACCTTGGAAGCCTACAGA
	*Il6*	CCCCAATTTCCAATGCTCTCC	AGGCATAACGCACTAGGTTT
	*Il13*	CTGCTACCTCACTGTAGCCT	TATTTCATGGCTGAGGGCTG
	*Il1* *7*	TCCACCGCAATGAAGACCCTGATA	ACCAGCATCTTCTCGACCCTGAAA
	*Il18*	AGGCATCCAGGACAAATCAG	GGTGTACTCATCGTTGTGGG
	*Il* *22*	CTTGTGCGATCTCTGATGGCT	GCTGGAAGTTGGACACCTCA
	*Il33*	TCCTGTCTGTATTGAGAAACCT	CTTATGGTGAGGCCAGAACG
	*Actb*	TGCTAGGAGCCAGAGCAGTA	AGTGTGACGTTGACATCCGT

## Data Availability

The data are included in the article.
